# Acetate as alternative carbon source for production of mono- and di-rhamnolipids in *Pseudomonas putida* KT2440

**DOI:** 10.1186/s12934-026-03050-6

**Published:** 2026-07-02

**Authors:** Jakob Grether, Sarah Leibinger, Christina Ramke, Philipp Hubel, Lisa Weber, Jens Pfannstiel, Elvio Henrique Benatto Perino, Rudolf Hausmann

**Affiliations:** 1 Department for Bioprocessing, University of Hohenheim, Stuttgart, Germany; 2 Core Facility, Mass Spectrometry Unit, University of Hohenheim, Stuttgart, Germany

**Keywords:** *Pseudomonasputida*, Rhamnolipid, Biosurfactant, Carbon source

## Abstract

**Background:**

The bacterium *Pseudomonas putida* KT2440 is used as a safe platform for rhamnolipid production via expression of the *rhlABC* genes, yielding mono- and di-rhamnolipid congeners. While glucose is commonly used as a carbon source, more sustainable alternatives such as acetate from waste streams or electrolysis are gaining interest. These two carbon sources are assimilated into the core carbon metabolism through distinct entry points. The impact of this on the proteome and rhamnolipid congener composition remain unclear. In this study, *P. putida* JAG1 was used to compare rhamnolipid production and composition during growth on glucose and acetate. Additionally, the impact of formate as a non-assimilable electron donor and the potential of modeled electrochemically derived CO₂-based carbon mixtures were evaluated.

**Results:**

Rhamnolipid precursor synthesis involves the rhamnose pathway and *de-novo* fatty acid synthesis. While glucose feeds directly into the rhamnose pathway and acetate into fatty acid synthesis, both carbon sources resulted in a similar di-rhamnolipid fraction of approximately 75 mol%. Although the carbon source significantly influenced the proteome profile, the carbon-to-nitrogen consumption ratio remained constant (C/*N* ≈ 15). Proteomic differences in *P. putida* JAG1 comparing the two carbon sources suggest ATP independent acetyl-CoA recycling as energy saving strategy. Enzyme abundances moreover suggest a coupling of the glyoxylate shunt and glycerol metabolism as strategy for gluconeogenesis. Growth rates, as well as biomass and product yields, were slightly lower on acetate compared to glucose. Co-feeding formate with acetate did not cause significant changes relative to acetate alone. Simultaneous consumption of acetate, formate, and ethanol only slightly affect product-per-biomass yields but strongly shifted the composition toward mono-rhamnolipids. Furthermore, comparison of the di-rhamnolipid producer with a mono-rhamnolipid producer indicates that di-rhamnolipid formation is associated with a higher molar product yield.

**Conclusions:**

Acetate resembles a viable alternative carbon source for rhamnolipid production with only slight drawbacks to glucose. The metabolism of the microbial cell factory *P. putida* JAG1 appears homeostatic in presence of a single assimilable carbon source without affecting the rhamnolipid congener composition. The availability of a second assimilable carbon source, however, shifted the composition in favor of the mono-rhamnolipid. The molar product yield per carbon atom was higher during production of di-rhamnolipids compared to sole production of mono-rhamnolipids, highlighting it as a promising target product for future studies.

**Supplementary Information:**

The online version contains supplementary material available at 10.1186/s12934-026-03050-6.

## Background

Biosurfactants, such as rhamnolipids, are ecofriendly molecules with, due to their versatile properties, diverse applications and increasing popularity in industrial biotechnology [1]. In this context, the heterologous production of rhamnolipids in *Pseudomonas putida* KT2440 has received a lot of attention during the last decades, and substantial work has been done for metabolic and process engineering [2].Glucose has long been regarded as a sustainable feedstock for biotechnology production. However, concerns have been growing because it is typically derived from agricultural crops, creating competition for arable land with food production and other land uses. In this context, the use of alternative carbon sources for the purpose of bioproduction has also been addressed, with the intention of finding viable alternatives to glucose. Acetic acid can be a future-oriented alternative carbon source for industrial biotechnology; recently high cell density bioprocesses based on lignocellulosic acetate as sole carbon source have been described [3, 4]. To date, acetic acid is mainly produced from fossil-based resources, and partly via fermentation with acetic acid bacteria. Newer approaches aim for the electrolytical conversion of CO_2_ and water to acetate. Electrolytically made acetate potentially has a negative carbon footprint, and its use for food and chemical production can significantly lower the cost [5]. Recently, it has been shown that the electrolysis product is a viable carbon source for the feeding of plants and algae, thereby replacing the natural process as artificial photosynthesis [6].

Rhamnolipid production in *Pseudomonas putida* KT2440 can be achieved through heterologous expression of the genes *rhlA*, *rhlB* and *rhlC*, which are derived from natural producers such as *P. aeruginosa* [7]. For biosynthesis, two types of precursor molecules must be generated by the cell. For the lipid moiety of the biosurfactant, 3-hydroxyalkanoic acid must be generated in fatty acid metabolism. The hydrophilic head consists of one or two rhamnosyl moieties deriving from glucose-6-phosphate (G6P), classifying the product as mono- or di-rhamnolipid, respectively. After its uptake, acetate is converted to acetyl-CoA, which directly feeds into *de-novo* fatty acid synthesis, yielding 3-hydroxydecanoic acid, the predominant lipid moiety of rhamnolipids [8]. In contrast, rhamnose moieties cannot be directly derived from acetyl-CoA and must instead be synthesized via gluconeogenesis, generating G6P and subsequently dTDP-rhamnose [7]. Glucose, on the other hand, is phosphorylated to G6P after its uptake and can directly feed into the rhamnose pathway. The conversion of glucose into acetyl-CoA for de novo fatty acid synthesis relies on the EDEMP cycle, a complex network that integrates the Embden-Meyerhof-Parnas, Entner-Doudoroff, and pentose phosphate pathways through a multitude of interconnected enzymatic reactions [9]. Accordingly, glucose and acetate enter rhamnolipid biosynthesis at distinct metabolic points in *P. putida*, each favoring either the rhamnose or fatty acid pathway, so precursor availability is expected to strongly depend on the carbon source. This metabolic separation provides a unique opportunity to use proteome analysis to determine how the entry of carbon sources into specific pathways affects the regulation of core metabolic enzymes that direct flux toward rhamnolipid biosynthesis. However, such comparative proteomic analysis has not been reported so far.

Rhamnolipid production on acetate was shown for mono-rhamnolipid production with product-to-substrate and product-per-biomass yields comparable to those of glucose [10]. The expression of *rhlA*, *rhlB and rhlC* in *P. putida* KT2440 using glucose as carbon source leads to production of a mixture of both mono-rhamnolipid (M-RL) and di-rhamnolipid (Di-RL), favoring the production of the Di-RL congeners [11, 12]. However, it is unclear whether the distinct metabolic entry points of glucose and acetate affect rhamnolipid product composition in *P. putida*.

The objective of this study was to examine the impact of utilizing glucose or acetate, in their respective capacities as sole carbon sources, on the heterologous production of M-RL and Di-RL, along with the ensuing effects on the proteome in chromosomally integrated strains of *P. putida* KT2440.

## Materials and methods

### Strains

To generate a plasmid-free rhamnolipid producer with high biosynthesis capability, two heterologous operons were integrated stepwise into *P. putida* KT2440 *Δupp*. First, a mono-rhamnolipid-producing strain was constructed by integrating a bi-cistronic *rhlAB* operon from *P. aeruginosa* PAO1 under control of the *rpsM* promoter into the intergenic region between *PP_0867* and *PP_0868*, yielding *P. putida* RAC10 (*Δupp*, P_rpsM_::*rhlAB*). For this construct, the *rpsM* promoter as well as the *yehX* and *PP_0867* homology regions were amplified from *P. putida* KT2440 genomic DNA, whereas *rhlA* and *rhlB* were amplified from *P. aeruginosa* PAO1 genomic DNA. All fragments were assembled in pJOE6261.2, and the resulting plasmid pCRA32 was used for markerless chromosomal integration via the *upp* counterselection system [13]. The full sequence of pCRA32 is provided in the supplementary materials (see Additional file 1).

For the second integration, *P. putida* RAC10 was used as the precursor strain for two independent chromosomal integrations at the attTn7 site via the mini-Tn7 delivery transposon vector pTn7-M [14]. In one approach, the *rhlCAB* operon was integrated, generating strain JAG1. In the second approach, an *rhlAB* operon was integrated, generating strain JAG2, each under control of the synthetic constitutive SynPro8 promoter [15]. For the construction of the *rhlCAB* delivery plasmid, *rhlC* was amplified from *P. aeruginosa* genomic DNA and inserted into pJG-rhlAB [12] by Gibson Assembly, yielding plasmid pJG-rhlCAB.

Subsequently, the SynPro8oT-*rhlCAB* and SynPro8oT-*rhlAB* operons were amplified from pJG-rhlCAB and pJG-rhlAB, respectively, and assembled into the pTn7-M backbone, resulting in pTn7-rhlCAB and pTn7-rhlAB. These plasmids were introduced into *P. putida* RAC10 via tetraparental mating, resulting in strains JAG1 and JAG2, respectively. While both strains, JAG1 and JAG2, have 2 copies of *rhlAB* integrated into the chromosome, JAG1 additionally has integrated one copy of *rhlC*.

All integrations were verified by Sanger sequencing. All constructed strains, plasmids and primers used for plasmid construction and verification are listed in the supplementary tables S1 and S2 (see Additional file 1).

### Media, sampling and cultivation conditions

#### General remarks

Unless otherwise stated, all chemicals used in this study were obtained from Carl Roth GmbH. All cultures were incubated at 30 °C at 120 rounds per minute (rpm) in an orbital shaker (New Brunswick™ Innova^®^ 44R, Eppendorf AG, Hamburg, Germany) with a shaking diameter of 5 cm. For M9 precultures and experimental cultures an initial working volume of 25 mL in 250 mL baffled shaking flasks was used.

### Media

Lysogeny Broth (LB) consisted of 5 g/L NaCl, 10 g/L tryptone and 5 g/L yeast extract. M9 medium consisted of 7.51 g/L Na_2_HPO_4_ × 2 H_2_O, 3 g/L KH_2_PO_4_, 0.25 g/L NaCl, 1 g/L (NH_4_)_2_SO_4_, 1 g/L MgSO_4_ × 7 H_2_O, pH 7.0 and trace elements with the following final concentrations:

6 × 10^− 3^ g/L FeSO_4_ × 7 H_2_O, 3.2 × 10^− 3^ g/L ZnSO_4_ × 7 H_2_O, 3.3 × 10^− 4^ g/L CuSO_4_ × 5 H_2_O,

3.7 × 10^− 4^ CoSO_4_ × 7 H_2_O, 8 × 10^− 5^ g/L H_3_BO_3_, 1.16 × 10^− 3^ g/L MnSO_4_ × H_2_O, 2.7 × 10^− 3^ g/L CaCO_3_. Glucose, sodium acetate, sodium formate and ethanol were added as indicated for each experiment. Sodium acetate and sodium formate were used to provide acetate or formate. For simplicity, the concentration is always expressed in terms of the salt’s anion.

### Sampling

Unless otherwise stated, cell supernatant was harvested by centrifugation for 15 min at 4 °C and 4700 × *g* and stored at -20 °C.

### Lysogeny broth precultures

For LB precultures, 20 µL of cryo-conserved *P. putida* JAG1 were used to inoculate 10 mL of LB containing 5 g/L NaCl, 10 g/L tryptone and 5 g/L yeast extract in 100 mL baffled shake flasks. The cultures were incubated for 24 h.

### M9 preculture

Unless otherwise stated, to prevent lag phases in experimental cultures, 250 µL of LB precultures were used to inoculate a second preculture of 25 mL M9 medium with 5 g/L acetate or glucose, in 250 mL baffled shake flasks. The cultures were incubated for 24 h and then used to inoculate experimental cultures.

### Comparison of acetate and glucose as sole carbon sources

The M9 preculture was used to inoculate M9 medium containing 3, 5 or 7 g/L glucose or sodium acetate, respectively, aiming for an initial optical density at 600 nm (OD_600_) of 0.2. Samples were taken regularly for OD_600_ determination. At the end of the growth phase, the culture supernatant was harvested and stored for later analysis. The number of replicates in this experiment were n_i_=3, n_j_=6 and n_k_=3 for 3 g/L, 5 g/L and 7 g/L initial carbon source concentrations, respectively.

### Co-feeding experiments with acetate and formate

The M9 preculture was used to inoculate M9 medium containing 5 g/L acetate with or without 0.9 g/L formate, respectively, aiming for an OD_600_ of 0.2. Samples were taken regularly and the culture supernatant was harvested and stored for later analysis. All cultivations were performed in triplicates.

### Carbon compound supplementation experiments

The M9 preculture was used to inoculate M9 medium containing a mixture of soluble carbon compounds derived from [16]. In one experiment the supplement concentration was 1 g/L of each acetate, ethanol and formate. In a different experiment, a composition of 1.6 g/L acetate, 1.0 g/L ethanol and 0.25 g/L formate was used. An initial OD_600_ of 0.1 was targeted for both experiments. Samples were taken regularly and the culture supernatant was harvested and stored for later analysis. All cultivations were performed in triplicates.

### Cell sampling for proteomics

For preculture preparation, 25 mL of LB in 250 mL baffled flasks were inoculated with 100 µL of *P. putida* JAG1 cryostock and incubated for 24 h at 30 °C and 120 rpm. Subsequently, 10 mL of preculture were withdrawn and divided equally into two 15 mL centrifuge tubes, one for each carbon source condition. To remove residual complex medium components, cells were harvested by centrifugation at 4 °C and 4700 × *g* for 6 min and each pellet was resuspended in 10 mL of M9 medium containing either 3 g/L acetate or glucose.

After resuspension, 1 mL of each cell suspension was used to inoculate five 100-mL flasks containing 10 mL M9 medium supplemented with the respective carbon source (3 g/L initial concentration), resulting in an initial OD_600_ of 0.25. Cultures were incubated at 30 °C and 120 rpm until mid-growth phase. Samples were taken when glucose-containing samples reached an OD_600_ of 1.4, and acetate-containing cultures, an OD_600_ of 1.2 (representing approximately the half maximum achievable biomass concentrations under these conditions).

For proteomic analysis, the whole cultures were centrifuged at 4 °C and 4700 × *g* for 7 min. The supernatant was discarded, and cell pellets were immediately frozen in -80 °C. Five biological replicates were prepared for each carbon source condition.

### LC-MS/MS proteomics sample preparation

#### Protein extraction for comparative proteome analysis

Cell pellets were thawed and suspended in lysis buffer containing 2% SDS (sodium dodecyl sulfate), 20 mM DTT (dithiothreitol) and 150 mM Tris-HCl pH 8.5 and incubated at 95 °C for 10 min. Cell lysates were cleared by centrifugation at 20.000 × g at 25 °C for 5 min. Proteins from the cleared lysates were precipitated by chloroform-methanol precipitation [17]. Protein pellets were resuspended in 6 M urea in 50 mM Tris-HCl pH 8.5 and protein concentrations were determined by a Bradford assay (Roti-Quant, Roth). Ten micrograms (10 µg) of the extracted proteins were used for in-solution digests. DTT (dithiothreitol) was added to a final concentration of 10 mM and incubated in an Eppendorf Thermomixer at 56 °C for 30 min. Reduced cysteines were subsequently alkylated by 40 mM CAA (chloroacetamide) for 30 min at room temperature (RT) in the dark. Alkylation was stopped by adding 50 mM DTT and samples were incubated for another 10 min at RT. Lysates were diluted with 50 mM Tris HCl pH 8.5 to a final concentration of 2 M urea and digested at 37 °C and 800 rpm for 16 h in an Eppendorf Thermomixer by adding 200 ng trypsin (protease to protein ratio of 1:50; Roche) and 100 ng LysC (protease to protein ratio of 1:100; Walko). TFA (trifluoroacetic acid) was added to the samples to a final concentration of 0.5%, esalted on C18 Stage Tips as described by Rappsilber et al. [18] and dried under vacuum. Dried samples were dissolved in 0.1% TFA.

### LC-MS/MS proteome analysis

NanoLC-MS/MS experiments were performed on an Ultimate 3000 nano-RSLC (Thermo Fisher Scientific, Germany) coupled to an Exploris 480 mass spectrometer (Thermo Fisher Scientific) using a Nanospray-Flex ion source (Thermo Fisher Scientific). Peptides were concentrated and desalted on a trap column (5 mm × 30 μm, Thermo Fisher Scientific, Dreieich, Germany) and separated on a 25 cm × 75 μm nanoEase MZ HSS T3 reversed phase column (100 Å pore size, 1.8 μm particle size, Waters, Milford, MA, United States of America) operated at constant temperature of 35 °C. Peptides were separated at a flow rate of 300 nL/min using a gradient with the following profile: 5% solvent B for 3 min, 5 − 55% solvent B in 60 min, 55 − 96% solvent B in 10 min, isocratic 96% solvent B for 10 min, 96% − 5% solvent B in 10 min and isocratic 5% solvent B for 10 min. The solvents used were 0.1% FA (solvent A) and 0.1% FA in ACN/H_2_O (80/20, v/v, solvent B). MS spectra (m/z = 300 − 1.600) were detected in the Orbitrap at a resolution of 60.000 (m/z = 200). The maximum injection time (MIT) for MS spectra was set to 50 ms, the automatic gain control (AGC) value was set to 3 × 10^6^. Internal calibration of the Orbitrap analyzer was performed using lock-mass ions from ambient air as described in Olsen et al. [19]. The MS was operating in the data-dependent mode selecting the top 20 highest abundant peptide precursor signals for fragmentation (HCD, normalized collision energy of 30%). For MS/MS analysis only precursor charge states from 2 to 6 were considered, the monoisotopic precursor selection was set to peptides, and the minimum intensity threshold was set to 1 × 10^5^. MS/MS scans were performed in the Orbitrap with a resolution of 15,000, isolation width was set to 1.6 Da. The AGC target was set to 5 × 10^4^, a max injection time was set to 50 ms and the first mass was set to 120 m/z. Dynamic exclusion was set to 60 s with a tolerance of 10 ppm.

### Proteomics MS data analysis

Raw files were imported into MaxQuant [20] version 2.0.1.0 for protein identification and label-free quantification (LFQ) of proteins. Protein identification in MaxQuant was performed using the database search engine Andromeda [21]. MS spectra and MS/MS spectra were searched against *P. putida* KT2440 sequence database and three additional sequences from *Pseudomonas aeruginosa* (UniProt ID: Q51559, Q51560, Q9I4K5) downloaded from UniProt [22]. Reversed sequences as decoy database and common contaminant sequences were added automatically by MaxQuant. Mass tolerances of 4.5 ppm (parts per million) for MS spectra and 20 ppm for MS/MS spectra were used. Trypsin was specified as enzyme and two missed cleavages were allowed. Carbamidomethylation of cysteines was set as a fixed modification and protein N-terminal acetylation and oxidation were allowed as variable modifications. The ‘match between runs’ feature of MaxQuant was enabled with a match time window of 0.7 minutes and an alignment time window of 20 minutes. Peptide false discovery rate (FDR) and protein FDR thresholds were set to 0.01.

MaxQuant output file (protein groups table) was loaded into Perseus version 1.6.14.0 [23]. LFQ values from MaxQuant were log2-transformed and matches to contaminants (e.g., keratins, trypsin) and reverse databases identified by MaxQuant were excluded from further analysis. Missing LFQ values were imputed in Perseus by replacing missing values from a normal distribution (Width = 0.3; Down shift = 1.8).

Significant changes in protein abundance between the acetate and glucose treated cells were analysed by a student’s t-test (two sided, S0 = 0) and corrected for multiple hypothesis testing using permutation-based FDR statistics (FDR = 0.05, 250 permutations).

The mass spectrometry proteomics data will be deposited to the ProteomeXchange Consortium [24] via the PRIDE [25] partner repository with the dataset identifier PXD074559.

### Quantifications

OD_600_ was measured using 0.9% NaCl as blank and diluent. Acetate, formate, ethanol, glucose and ammonia concentrations were all measured using enzymatic assays from Enzytec™, R-Biopharm AG, Darmstadt, Germany.

Rhamnolipid quantification was performed according to [15] with slight adjustments as follows. For rhamnolipid analysis, 2 mL of cell free supernatant obtained from end point samples, were acidified with 20 µL of 85% *ortho*-phosphoric acid and extracted three times with 2.5 mL of ethyl acetate. The three extracted organic phases obtained from each sample were pooled and dried in a vacuum centrifuge (2 h, 45 °C, 10 mBar). The remaining rhamnolipid extracts were dissolved in 2 mL of acetonitrile. Quantification is based on fluorescence extinction, which is mediated via rhamnolipid derivatization to form *p*-bromophenacyl esters, as described before [12, 26]. For this, 9 volumes of extract were mixed with 1 volume of derivatizing agent (1:1-mixture (v/v) of 135 mM 2,4-Dibromoacetophenone and 67.5 mM Tri-ethylamin) and incubated (60 °C, 1600 rpm). Rhamnolipid (99% purity) obtained from former company Hoechst served as standard. Derivatized samples and standards were applied on a fluorescent HPTLC silica gel 60 F254 plate (VWR). Plates were developed using a volumetric mixture of isopropyl acetate, ethanol, water and glacial acetic acid in volumetric ratios 1 : 0.45 : 0.08 : 0.03. Separation of mono- and di-rhamnolipids according to their hydrophobicity on the HPTLC plate allowed separate quantification. The calibration curve comprised 8 different amounts of rhamnolipid (0.075 µg, 0.125 µg, 0.25 µg, 0.625 µg, 1 µg, 1.5 µg, 2 µg and 2.5 µg). For a depiction of rhamnolipid separation by HPTLC and the standard calibration curves, see Additional file 4.

Yield coefficients and C/N-ratio were determined over the whole cultivation, as specified in Eq. (1) for Y_XS_, Eq. (2) for Y_PX_ and Eq. (3) for the atomic C/N-ratio with *α* being the number of atoms per molecule of carbon source, i.e. 2 for acetate and 6 for glucose.

1 $$\:\mathrm{S}\mathrm{p}\mathrm{e}\mathrm{c}\mathrm{i}\mathrm{f}\mathrm{i}\mathrm{c}\:\mathrm{b}\mathrm{i}\mathrm{o}\mathrm{m}\mathrm{a}\mathrm{s}\mathrm{s}\:\mathrm{y}\mathrm{i}\mathrm{e}\mathrm{l}\mathrm{d}\:{Y}_{XS}=\frac{{\varDelta\:m}_{biomass}}{{\varDelta\:m}_{carbon\:source}}$$

2 $$\:\mathrm{S}\mathrm{p}\mathrm{e}\mathrm{c}\mathrm{i}\mathrm{f}\mathrm{i}\mathrm{c}\:\mathrm{p}\mathrm{r}\mathrm{o}\mathrm{d}\mathrm{u}\mathrm{c}\mathrm{t}\:\mathrm{y}\mathrm{i}\mathrm{e}\mathrm{l}\mathrm{d}\:{Y}_{PX}=\frac{{\varDelta\:m}_{(Di-RL+M-RL)}}{{\varDelta\:m}_{biomass}}=\frac{{\varDelta\:m}_{RL}}{{\varDelta\:m}_{biomass}}$$

3 $$\:\frac{\mathrm{C}}{\mathrm{N}}-\mathrm{r}\mathrm{a}\mathrm{t}\mathrm{i}\mathrm{o}\:\:C/N=\raisebox{1ex}{$\frac{\alpha\:*{\varDelta\:m}_{carbon\:source}}{{M}_{carbon\:source}}$}\!\left/\:\!\raisebox{-1ex}{$\frac{{\varDelta\:m}_{ammonia}}{{M}_{ammonia}}$}\right.$$

For calculation of the molar concentrations and fractions of the rhamnolipid congeners, an acyl chain length of C10 was anticipated for the mono- (504 g/mol) and di-rhamnolipid (650 g/mol) congeners.

An estimate of the biomass cell dry weight was calculated from OD_600_ using a conversion factor of 0.26 (g/L)/ OD_600_.

The average specific growth rates (µ) were determined by plotting the natural logarithm of the OD₆₀₀ values measured during the exponential growth phase against the culture time.

### Generation of metabolic maps

The log2 converted fold changes (log2(fc)) of protein abundances calculated from mass spectrometry data were used for representation of the metabolic regulation. For this, reactions were numbered and enzymes involved in the reactions were grouped. If several enzymes were known to catalyze the same reaction, the highest or lowest value was used for the representation in the metabolic map, marked in red for downregulation and blue for upregulation. If for one reaction several enzyme isoforms with both up- and downregulation, were observed, both directions of regulation were indicated with two-color code. An increased or decreased protein abundance of ≥ 20% (which is a log2(fc) of ± 0.263) was defined as regulation. Regulation with no statistical significance is indicated on the map using a not-equal-sign (≠). The map was created manually using MS Powerpoint.

## Results

### Effect of carbon sources on strain performance

To investigate the conversion of acetate and glucose to biomass and rhamnolipid, *P. putida* JAG1 was cultivated in shake flasks, spanning initial carbon source concentrations of 3, 5 and 7 g/L. Biomass-per-substrate yield Y_XS_ and Product-per-biomass yield Y_PX_ was higher for glucose than for acetate. Growth rates were higher on glucose. For both carbon sources, the consumption of carbon per nitrogen was identical with a usage ratio (C/N ratio) of approximately 15 (Table 1). The data used for quantification is provided (see Additional file 2).

**Table 1:** Results of shake flask cultivations of *P*.*putida* JAG1 with glucose or acetate as sole carbon source

	µ^average^ [1/h]			C/*N* ratio [C-mol/*N*-mol]	Y_XS_ [g_CDW_/g_substrate_]	Y_PX_ [mg_RL_/g_CDW_]
	c^init^ 3 g/L	c^init^ 5 g/L	c^init^ 7 g/L			
Acetate	0.38 ± 0.01	0.38 ± 0.02	0.36 ± 0.01	14.90 ± 2.09	0.17 ± 0.02	176.98 ± 25.35
Glucose	0.47 ± 0.01	0.47 ± 0.00	0.44 ± 0.00	15.03 ± 3.01	0.27 ± 0.02	204.28 ± 22.09

Growth rates µ were determined from linear regression. The C/N ratio represents the ratio of consumed carbon and nitrogen atoms. Y_XS_ and Y_PX_ were calculated as average and standard deviation from all 12 biological replicates. For Y_PX_, mono- and di-rhamnolipid were summed

**Fig. 1:**
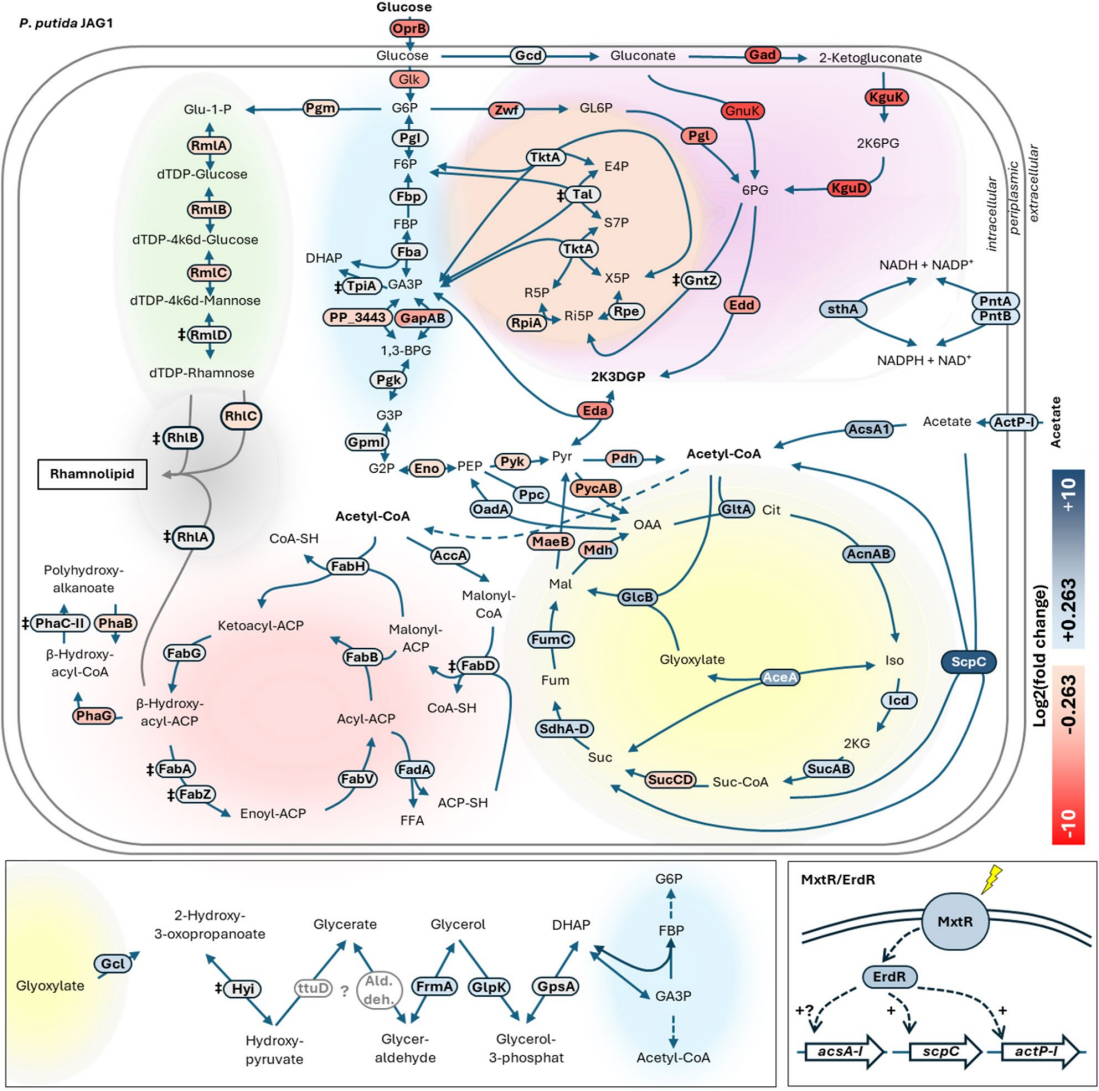
Heatmap of proteome analysis of *P. putida* JAG1 using glucose or acetate as sole carbon source

Independent of the carbon source, a mixture of di-rhamnolipids and mono-rhamnolipid was produced, and the amount of di-rhamnolipid was always higher than the amount of mono-rhamnolipid. Comparing the rhamnolipid yield per carbon atom, it was higher when glucose was used as carbon source for both M-RL and Di-RL. With the two carbon sources used, the pathways for dTDP-rhamnose production are different with respect to the number of reactions, since from acetate, gluconeogenesis must happen, while glucose can more directly be used (compare Fig. 1). Hypothetically, this might lead to different dTDP-rhamnose availability and finally, to a different product ratio when using these two carbon sources. Interestingly, the relative number of di-rhamnolipid molecules per total rhamnolipid molecules did not differ between the two carbon sources. Data are summarized in Table 2. In contrast to glucose consumption, which did not markedly affect the pH, acetate consumption resulted in an increase in pH to approximately 9.0. The rhamnolipid yield per carbon atom was relatively stable over the whole range of glucose concentration. In the case of acetate, however, substrate consumption was associated with a decrease in the rhamnolipid yield per carbon atom, possibly linked to the concomitant increase in pH (see Additional file 2).

**Table 2:** Substrate conversion and product composition with glucose or acetate as sole carbon source

Carbon source	M-RL yield	Di-RL yield	M-RL per carbon	Di-RL per carbon	Molar Di-RL fraction
	$\:\left[\frac{{\boldsymbol{\upmu\:}\mathbf{m}\mathbf{o}\mathbf{l}}^{\mathbf{M}-\mathbf{R}\mathbf{L}}}{{\mathbf{m}\mathbf{m}\mathbf{o}\mathbf{l}}^{\mathbf{s}\mathbf{u}\mathbf{b}\mathbf{s}\mathbf{t}\mathbf{r}\mathbf{a}\mathbf{t}\mathbf{e}}}\right]$	$\:\left[\frac{{\boldsymbol{\upmu\:}\mathbf{m}\mathbf{o}\mathbf{l}}^{\mathbf{D}\mathbf{i}-\mathbf{R}\mathbf{L}}}{{\mathbf{m}\mathbf{m}\mathbf{o}\mathbf{l}}^{\mathbf{s}\mathbf{u}\mathbf{b}\mathbf{s}\mathbf{t}\mathbf{r}\mathbf{a}\mathbf{t}\mathbf{e}}}\right]$	$\:\left[\frac{{\boldsymbol{\upmu\:}\mathbf{m}\mathbf{o}\mathbf{l}}^{\mathbf{M}-\mathbf{R}\mathbf{L}}}{{\mathbf{m}\mathbf{m}\mathbf{o}\mathbf{l}}^{\mathbf{C}-\mathbf{a}\mathbf{t}\mathbf{o}\mathbf{m}\mathbf{s}}}\right]$	$\:\left[\frac{{\boldsymbol{\upmu\:}\mathbf{m}\mathbf{o}\mathbf{l}}^{\mathbf{D}\mathbf{i}-\mathbf{R}\mathbf{L}}}{{\mathbf{m}\mathbf{m}\mathbf{o}\mathbf{l}}^{\mathbf{C}-\mathbf{a}\mathbf{t}\mathbf{o}\mathbf{m}\mathbf{s}}}\right]$	$\:[\frac{{\mathbf{m}\mathbf{o}\mathbf{l}}^{\mathbf{D}\mathbf{i}-\mathbf{R}\mathbf{L}}}{{\mathbf{m}\mathbf{o}\mathbf{l}}^{\boldsymbol{R}\boldsymbol{L}}},\boldsymbol{\%}]$
Acetate	0.79 ± 0.23	2.14 ± 0.40	0.40 ± 0.12	1.07 ± 0.20	73.11 ± 3.67
Glucose	4.05 ± 0.52	11.91 ± 1.43	0.68 ± 0.09	1.98 ± 0.24	74.62 ± 1.08

Values are calculated from all 12 biological replicates. Molar weights of the main rhamnolipid congeners (10 carbon atoms per fatty acid chain) were used (mono-rhamnolipid: 504 g/mol; di-rhamnolipid: 650 g/mol)

### Proteome analysis

To get insights in differential usage of these two carbon sources, proteomes were compared during exponential growth using acetate or glucose, respectively, as sole carbon sources. The effect on the core carbon metabolism is presented in Fig. 1.

The differences in protein abundance in the utilization of acetate compared to glucose are shown in the color code as log2 converted fold change. Enzymes marked in red are downregulated in the presence of acetate as the only carbon source, enzymes marked in blue are upregulated. Enzymes marked in two colors represent isoforms with opposing changes in abundance. Enzymes marked in white were not regulated. Main: Central and rhamnolipid metabolism; bottom left: Proposed gluconeogenetic pathway via glycerol; bottom right: MxtR/ErdR-2-component system (the lightning represents a trigger activating MxtR, which in turn activates ErdR; ‘+’ indicates positive regulation of target genes by ErdR). Non-significant differences are highlighted with ‘‡’.

During growth on acetate, enzymes of the initial glucose catabolism via the Entner-Doudoroff pathway (pink) were strongly downregulated, while enzymes of the Embden-Meyerhof-Parnas (blue) were only slightly affected. There were no differences in regulations on the pentose phosphate pathway (brown). The most enzymes of the citric cycle and glyoxylate shunt (both in yellow) were upregulated. Here, the strongest upregulation was observed for succinyl: CoA acetate transferase (ScpC), isocitrate lyase (AceA) and malate synthase (GlcB). Interestingly, the abundance of enzymes involved in DHAP formation from the glyoxylate shunt increased. These enzymes include glyoxylate carboligase (Gcl) and glycerol kinase (GlpK). Conversely, the abundance of enzymes linked to DHAP formation from pyruvate decreased, including the pyruvate carboxylases PycA and PycB, enolase (Eno), and in particular, 2-keto-3-deoxy-6-phosphogluconate aldolase (Eda).

Phosphoenolpyruvate carboxylase (Ppc) and oxaloacetate decarboxylase (OadA), which catalyze reactions with opposing direction, were both upregulated. Both proteins of the MxtR/ErdR 2-component system (Fig. 1, bottom right), which is a key player in acetate catabolic control, were upregulated under exposure to acetate as sole carbon source.

In fatty acid synthesis (red), different conditions had little effect on enzyme abundance. PhaG, which catalyzes the first reaction in the production of the competing by-product polyhydroxyalkanoate (PHA) was downregulated during growth on acetate. Abundance of enzymes of the dTDP-rhamnose pathway (green) was slightly reduced. Enzyme abundance of constitutively expressed heterologous rhamnolipid synthesis genes *rhlA*, *rhlB* and *rhlC* (grey) were almost unaffected, however RhlC was slightly less abundant. The full data set of the proteomic analysis is provided in the supplementary material (see Additional file 3).

### Evaluation of formate as co-substrate during acetate consumption

Growth on acetate resulted in decreased yields (Y_XS_, Y_PX_) compared to glucose, which may be related to a lower degree of NAD(P)H regeneration during growth on the small organic acid. *P. putida* KT2440 is naturally equipped with formate dehydrogenase, which converts formate to CO_2_ by regeneration of NADH from NAD^+^. Previously it has been shown that the co-consumption of glucose and formate elevates the NADH regeneration rate in *P. putida*, which resulted in increased anabolism [27]. To investigate the effects of co-feeding formate on growth and rhamnolipid production, *P. putida* JAG1 and the M-RL producing strain *P. putida* JAG2 were cultivated comparatively on acetate and a mixture of acetate and formate, respectively.

Co-feeding of *P. putida* JAG1 with acetate and formate had no considerable effect, neither on Y_XS_ nor on Y_PX_ under these conditions. The relative molar share of Di-RL was unaffected by the presence of formate. Notably, comparing the two strains under similar initial conditions, the total molar rhamnolipid yield was always higher for the *P. putida* JAG1, although both strains contained the same number of operons with identical structure, despite the lack of the *rhlC* gene in *P. putida* JAG2. The results of the co-feeding are summarized in Table 3. Cultivation data are provided in the supplementary data (see Additional file 2).

**Table 3:** Yield comparison of cultivations with *P. putida* strains JAG1 and JAG2

Carbon compounds	*P*. putida JAG1		*P*. putida JAG2	
	Acetate	Acetate + formate	Acetate	Acetate + formate
Y_xs_ [g_CDW_/ g_acetate_**]**	0.18 ± 0.01	0.16± 0.00	0.21 ± 0.01	0.21± 0.00
Y_px_ [mg_RL_/g_acetate_**]**	162.84 ± 10.00	154.5± 4.99	78.83 ± 3.20	81.91± 6.63
Di-RL fraction $\:[\frac{{\boldsymbol{m}\boldsymbol{o}\boldsymbol{l}}^{\boldsymbol{D}\boldsymbol{i}-\boldsymbol{R}\boldsymbol{L}}}{{\boldsymbol{m}\boldsymbol{o}\boldsymbol{l}}^{\boldsymbol{t}\boldsymbol{o}\boldsymbol{t}\boldsymbol{a}\boldsymbol{l}\:\boldsymbol{R}\boldsymbol{L}}},\:\boldsymbol{\%}]$	77.74 ± 1.09	77.84± 0.74	not applicable	not applicable
Molar RL yield $\:\left[\frac{{\boldsymbol{\mu\:}\boldsymbol{m}\boldsymbol{o}\boldsymbol{l}}^{\boldsymbol{R}\boldsymbol{L}}}{{\boldsymbol{m}\boldsymbol{m}\boldsymbol{o}\boldsymbol{l}}_{\boldsymbol{a}\boldsymbol{c}\boldsymbol{e}\boldsymbol{t}\boldsymbol{a}\boldsymbol{t}\boldsymbol{e}}^{\boldsymbol{C}-\boldsymbol{a}\boldsymbol{t}\boldsymbol{o}\boldsymbol{m}\boldsymbol{s}}}\right]$	1.43 ± 0.07	1.21± 0.04	0.97 ± 0.04	1.00 ± 0.04

### Evaluation of rhamnolipid production in model electrolysis medium

**Fig. 2:**
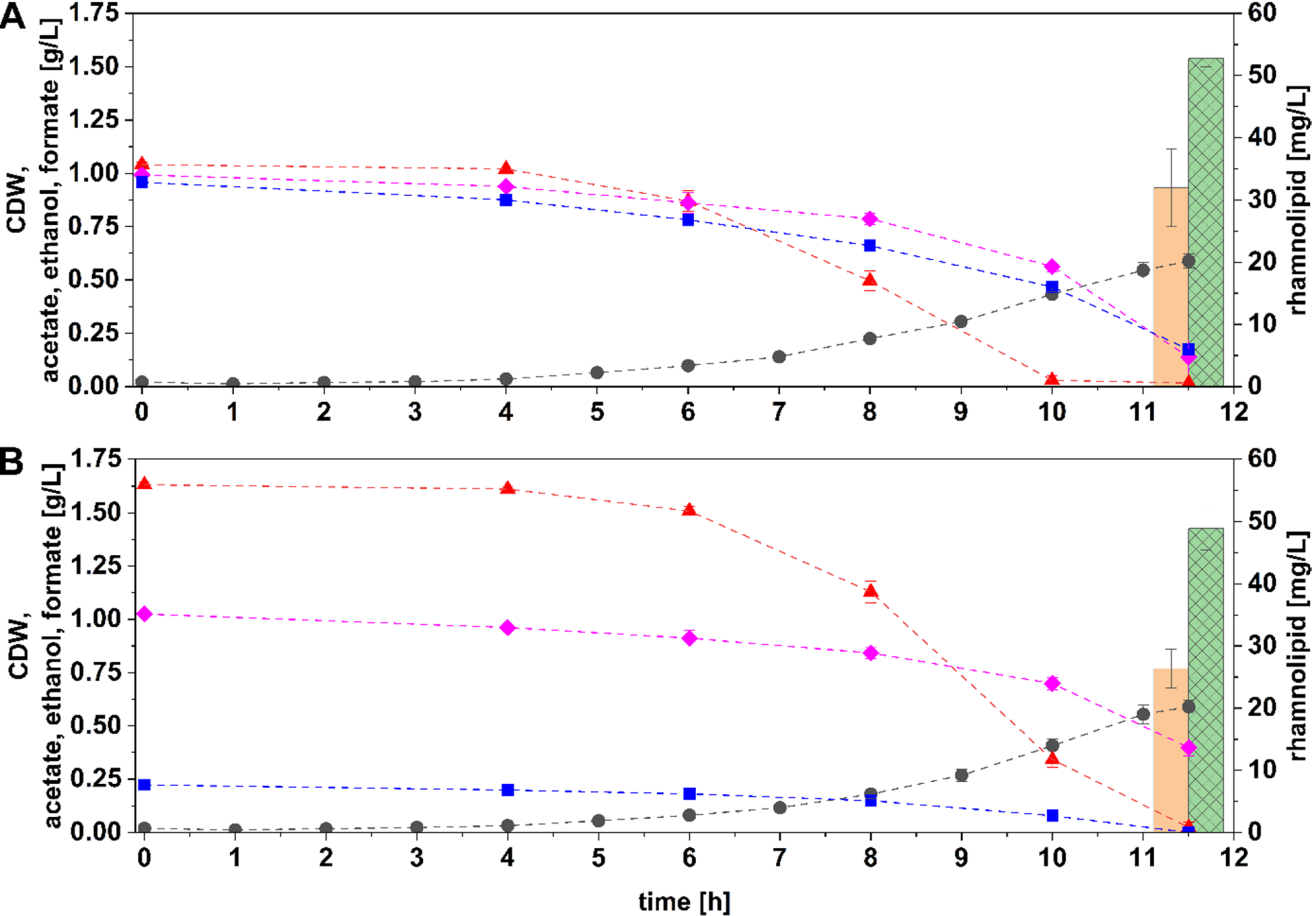
Time course of co-feeding experiments of *P. putida* JAG1 with model electrolysis products. The media contained a mixture of acetate (red triangles), ethanol (purple diamonds) and formate (blue squares). Cell dry weight (CDW) is shown as black dots, mono-rhamnolipid and di-rhamnolipid are shown as brown or green hatched bars, respectively. **A**: Course of cultivation in a medium with approximately 1 g/L initial carbon compound concentration. **B**: Course of cultivation in a medium with 1.6 g/L acetate, 1 g/L ethanol and 0.25 g/L formate

Electrolysis offers a promising route for reducing CO₂ to acetate. However, acetate production via electrolysis is typically accompanied by side products such as ethanol and formate. While ethanol can serve as an additional assimilable carbon source for *P. putida*, formate is not assimilated but may be used for NADH regeneration. To investigate rhamnolipid production on CO_2_-derived carbon compounds containing two assimilable carbon sources, namely acetate and ethanol, M9 medium was supplemented with representative mixtures of the carbon compounds. In one experiment, each carbon compound was supplied at 1 g/L to determine the preferred carbon source. In a second experiment, representative concentrations were derived from literature, with acetate being the main compound [16]. Both model media were inoculated with *P. putida* JAG1 (Fig. 2).

In the model electrolysis product with 1 g/L acetate, ethanol and formate, all carbon components were consumed simultaneously, however with different rates, with acetate being depleted most rapidly (after 10 h). Ethanol and formate were consumed slower and in almost similar rates, with residual concentrations of 0.14 and 0.17 g/L, respectively, at the end of the cultivation (11.5 h). Mono- and di-rhamnolipid titers were 31.93 ± 6.24 mg/L and 52.78 ± 1.32 mg/L, respectively, representing 56.17 ± 4.85 mol% of Di-RL per total RL. Y_PX_ was 149.14 ± 15.80 mg_RL_/g_CDW_.

In the model electrolysis product containing acetate as the main carbon compound, acetate and formate were consumed after 11.5 h. A residual ethanol concentration of 0.4 g/L was observed at that time point. Final mono- and di-rhamnolipid titers were 26.36 ± 3.14 mg/L and 48.90 ± 3.44 mg/L, respectively, representing 58.99 ± 3.35 mol% of Di-RL per total RL. Y_PX_ was 132.10 ± 13.94 mg_RL_/g_CDW_. The cultivation dataset is provided in the supplementary data (see Additional file 2). In addition, a control experiment was performed to exclude abiotic effects, such as volatility, as a cause of the observed decrease in carbon source concentration (see Additional file 5).

## Discussion

The use of glucose in industrial biotechnology is increasingly criticized for reasons of reliance on land and competition with the human food chain. Consequently, new sources for more sustainable carbon sources with comparable costs and value for bioprocessing are brought into focus. The efficient use of acetate as carbon source for bioproduction has been shown for several value-added chemicals [28]. A novel approach for acetate synthesis is the electrolysis of CO_2_ and water. Electro-synthesized acetate is promising as a future carbon source because production costs for food and chemicals, as well as price fluctuations, are reduced compared to glucose [16, 29]. The use of acetate as carbon source for bioproduction bears high potential, since after its uptake, it is converted to acetyl-CoA, which is a turnstile molecule and therefore gateway for many synthesis pathways. A previous study has shown that mono-rhamnolipid production in a plasmid-based *P. putida* production strain using acetate as the sole carbon source was comparable to glucose [10].

### Effect of carbon sources on rhamnolipid congener composition

Rhamnolipid biosynthesis requires precursors from two central metabolic pathways: glucose-6-phosphate for the rhamnose moiety and acetyl-CoA for the formation of hydroxyalkanoyloxy alkanoic acid (HAA) via fatty acid synthesis [7, 8]. Glucose and acetate therefore provide different metabolic entry points: glucose directly supports rhamnose precursor formation, while acetate directly supplies acetyl-CoA. In *P. putida* KT2440, the heterologous expression of *rhlABC* results in a mixture of mono- and di-rhamnolipids, with di-rhamnolipids typically being the dominant product [11, 12]. Since both precursor pathways are directly linked to the supplied carbon source, this study investigates whether the choice of carbon source affects the ratio of mono- to di-rhamnolipids and how the proteome of the microbe is affected by the presence of either acetate or glucose. The plasmid-free producer strain *P. putida* JAG1 efficiently utilized the small organic acid acetate, although growth rate and yield coefficients were lower than those observed with glucose (Table 1). This difference can be explained by the lower amount of reducing power generated during acetate catabolism. In *P. putida* KT2440, glucose is metabolized via the tripartite EDEMP pathway, which provides an efficient route for NAD(P)H generation. In contrast, acetate is first converted to acetyl-CoA and enters central metabolism at a more oxidized metabolic entry point. Moreover, acetate consumption is accompanied by an equimolar uptake of protons, resulting in an increase in pH, which may impose additional constraints on microbial homeostasis. The consumption of acetate resulted in an increase in pH from 7.0 to approximately 9.0, while glucose consumption did not affect the culture pH. Although *P. putida* is relatively tolerant to withstand acetate concentrations up to 10 g/L, it is likely that toxic effects are already affecting the bacterial fitness in lower concentrations [30]. As the amount of acetate consumed increased, the rhamnolipid yield per carbon atom decreased, leading to generally greater uncertainty in the quantification when acetate was used as the carbon source (see Additional file 2). For the use of acetate as carbon source in large scale bioproduction processes, monitoring and maintaining optimal pH values is therefore crucial to allow high productivity. Although the carbon sources enter the central metabolism at different points, the molar C/N ratio of substrate uptake was 15 on both acetate and glucose, indicating a conserved stoichiometry of carbon and nitrogen uptake. While this may be consistent with a stable biomass composition, differences in carbon oxidation and CO₂ evolution cannot be excluded (Table 1). The tight coupling between carbon and nitrogen uptake reflects a high degree of metabolic homeostasis and might also explain the conserved ratio of mono- and di-rhamnolipid congeners produced by *P. putida* JAG1, independent of the investigated carbon source. Despite the different pathways, each of which is directly fed by one of the two carbon sources, the di-rhamnolipids make up approximately 75% of all rhamnolipid molecules in both cases (Table 2). Also, a supplementation of formate to acetate-containing medium did not change the Di-RL fraction (Table 3). Besides the homeostatic character of the *P. putida* metabolism, this might be explained by a high intracellular availability of precursor molecules that are not fully converted due to the limited production capacity of the producer strain. However, when ethanol and formate were supplemented to simulate an electrolysis-derived substrate mixture, the composition of the rhamnolipid congeners in the product changed significantly, with a shift towards M-RL, while the product-per-biomass yield was slightly decreased compared with the acetate-only cultivation (Fig. 2). This observation suggests that changes in the availability of assimilable carbon sources may influence the allocation of precursors and, consequently, the composition of rhamnolipid congeners. Future studies, ideally including metabolic flux analysis, should therefore investigate whether the type and combination of assimilable carbon sources induce a rerouting of central carbon fluxes toward specific RL precursors. At the same time, the non-diauxic consumption of acetate, ethanol and formate points out the suitability of *P. putida* KT2440 as microbial production host for investigating the biotechnological use of electrolysis-derived carbon sources.

Compared to glucose, using acetate as sole carbon source allows for lower growth rates and yields, which may be partly associated with lower NADH regeneration. In a previous study, co-feeding of formate and glucose in continuous bioreactor cultivations resulted in increased biomass yield coefficients of *P. putida* KT2440 [27]. In the present study, co-feeding of acetate and formate did not affect the yield coefficients of *P. putida* JAG1 or JAG2, however the results were obtained from shaking flask cultures, and biomass was estimated from OD₆₀₀ using a conversion factor, rather than determined gravimetrically. Therefore, biomass-related yield coefficients represent estimates based on OD-derived biomass and may be affected by changes in cell morphology or by the formation of intracellular storage compounds, such as polyhydroxyalkanoates (PHAs), particularly under nitrogen-limited conditions [31]. To better evaluate the effect of acetate and formate co-consumption on biomass-related yield coefficients (Y_PX_ and Y_XS_), continuous bioreactor cultivations would be required.

The high share of approximately 75 mol% Di-RL in the culture supernatant of *P. putida* JAG1 was surprising, since this strain only contains a single copy of the *rhlC* gene but two copies of both the *rhlA* and *rhlB* genes. This indicates a high reaction rate of RhlC. Moreover, even though *P. putida* JAG1 and JAG2 carry the same number of rhamnolipid operons under control of the same promoters, the molar RL yield per carbon atom was higher for the Di-RL producer (1.43 ± 0.07 µmol^RL^/mmol^C−atoms^) than for the M-RL producer (0.97 ± 0.04 µmol^RL^/mmol^C−atoms^) (Table 3). This uncovers the potential of *P. putida* KT2440 to be a more potent producer of Di-RL, while it is mostly used for production of M-RL so far. Possibly, the secretion of Di-RL is easier than of M-RL. It has been shown that in *P. aeruginosa* PAO1 the membrane-bound protein EstA promotes rhamnolipid secretion [32]. Although there is no homologue protein in *P. putida* KT2440, another protein might exist which specifically promotes the secretion of the Di-RL congeners. An efficient secretion of Di-RL could therefore decrease intracellular M-RL concentrations and increase the flux through the rhamnolipid synthesis pathways to the final product, similar to the mechanism described for the use of strong promoters in M-RL producing *P. putida* strains [33].

### Adaptation of the *P. putida* proteome to acetate as sole carbon source

To gain insight into how the two carbon sources influence the metabolism of *P. putida* KT2440, proteome analysis was performed. A quantitative assessment of intracellular carbon fluxes would require ^13^C-based metabolic flux analysis. However, this approach relies on specialized analytical equipment and was therefore beyond the scope of this study. Nevertheless, proteome analysis can provide valuable insights into condition-dependent changes in metabolic pathway capacity and cellular resource allocation, thereby offering an approximate systems-level indication of metabolic activity. This is reflected, for example, by the abundance of glucose metabolic enzymes in the presence of acetate as the sole carbon source, and vice versa (Fig. 1). In the case of glucose, after it is taken up into the periplasm, its degradation pathway is divided into three convergent pathways that simultaneously feed the Embden-Meyerhof-Parnas (EMP), Entner-Doudoroff (ED), and pentose phosphate (PP) pathways, a situation that was termed the EDEMP cycle [9]. In proteome analysis HexR, the main transcription regulator for glucose metabolism pathways [34], was downregulated during growth on acetate. This is reflected in the lower abundance of enzymes, especially of the ED pathway (Fig. 1). Quantitative metabolic flux analyzes found this pathway as main metabolic route during glucose consumption in *P. putida*, thereby producing a surplus of NADPH [9, 35].

The metabolization of acetate is initiated by activation of the MxtR/ErdR two-component system, which has been determined to be essential for the utilization of acetate in *P. putida* KT2440 by transactivation of *scpC* and *actP-I* expression [36]. In the present proteome analysis, both components were significantly upregulated in presence of acetate as sole carbon source with log2(fc) = 2.21 for ErdR (PP_1635) and log2(fc) = 1.75 for MxtR (PP_1695). In congruence with this, the acetate transporter ActP-I was slightly upregulated (log2(fc) = 0.35) when acetate was consumed as the sole carbon source. After acetate is taken up into cytoplasm, it is further activated to form acetyl-CoA, from where it is guided towards the tricarboxylic acid cycle and fatty acid anabolism. For the ATP-dependent activation of acetate, several candidate enzymes come into question, namely the acyl-CoA synthetase Acs (PP_3458) as well as the acetyl-CoA synthetases AcsA-I (PP_4487) and AcsA-II (PP_4702). Among these, only AcsA-I was upregulated during acetate consumption (log2(fc) = 2.47), suggesting Acs and AcsA-II as less important for *P. putida* KT2440 under the experimental conditions. The succinyl-CoA: acetate CoA transferase ScpC (PP_0154) was strongly upregulated during growth on acetate (log2(fc) = 9.44), highlighting its importance for growth under these conditions. This enzyme facilitates the rapid conversion acetate into acetyl-CoA by transferring CoA from succinyl-CoA to acetate. This mechanism has been described for the high acid tolerant bacterium *Acetobacter acetii*, which expresses AarC [37]. Deletion of *aarC* in *A. acetii* strongly impeded growth on acetate as sole carbon source [38]. Besides this alleviation of toxic intracellular acetate levels, *scpC* expression presumably represents a compensatory strategy, enabling ATP-independent acetate activation via CoA transfer from succinyl-CoA and thereby partially offsetting the energetic costs associated with acetate utilization.

This adaption of the TCA cycle might allow a rapid regeneration of NADH by using a shortcut pathway between succinyl-CoA an acetate, thereby generating succinate independent of the ATP-using reaction of SucCD. This hypothesis is supported by upregulation of every TCA cycle enzyme except SucCD, which was downregulated (log2(fc)=-0.87). Taken together, the main reactions for acetate activation of *P. putida* might be realized by AcsA-I and ScpC, however this needs further investigation by complementary methods such as ^13^C metabolic flux analysis.

To produce rhamnolipid from acetate as sole carbon source, gluconeogenesis is required to generate the sugar moieties of the product. For this, 2 pathways come into question: Either, oxaloacetate (OAA) can be decarboxylated by OadA to generate phosphoenolpyruvate (PEP) as a starting point for a reversed glycolytic pathway together with enzymes of the EDEMP pathway to generate glucose-6-phosphate [39]. Abundance of OadA was slightly increased (log2(fc) = 0.28). At the same time, the abundance of Ppc, which has opposite reaction direction (producing OAA from PEP), was also slightly increased (log2(fc) = 0.62), while neighboring enzymes Eno (log2(fc)=-0.32) and Pyk (log2(fc)=-0.62) were downregulated, questioning if this metabolic node is the route for gluconeogenesis. Another possibility is the reduction conversion of glyoxylate to glycerol, which is then further metabolized to glycerol-3-phosphate and finally DHAP. Although in this pathway some enzymes (catalyzing the reactions hydroxypyruvate → glycerate ↔ glyceraldehyde) were not detected, key enzyme reactions with thermodynamic constraint in forward direction were upregulated (glyoxylate carboligase, PP_4297, log2(fc) = 1.17; glycerol kinase, PP_1075, log2(fc) = 0.32), suggesting this as an alternative pathway for gluconeogenesis (Fig. 1, bottom left).

**Fig. 3:**
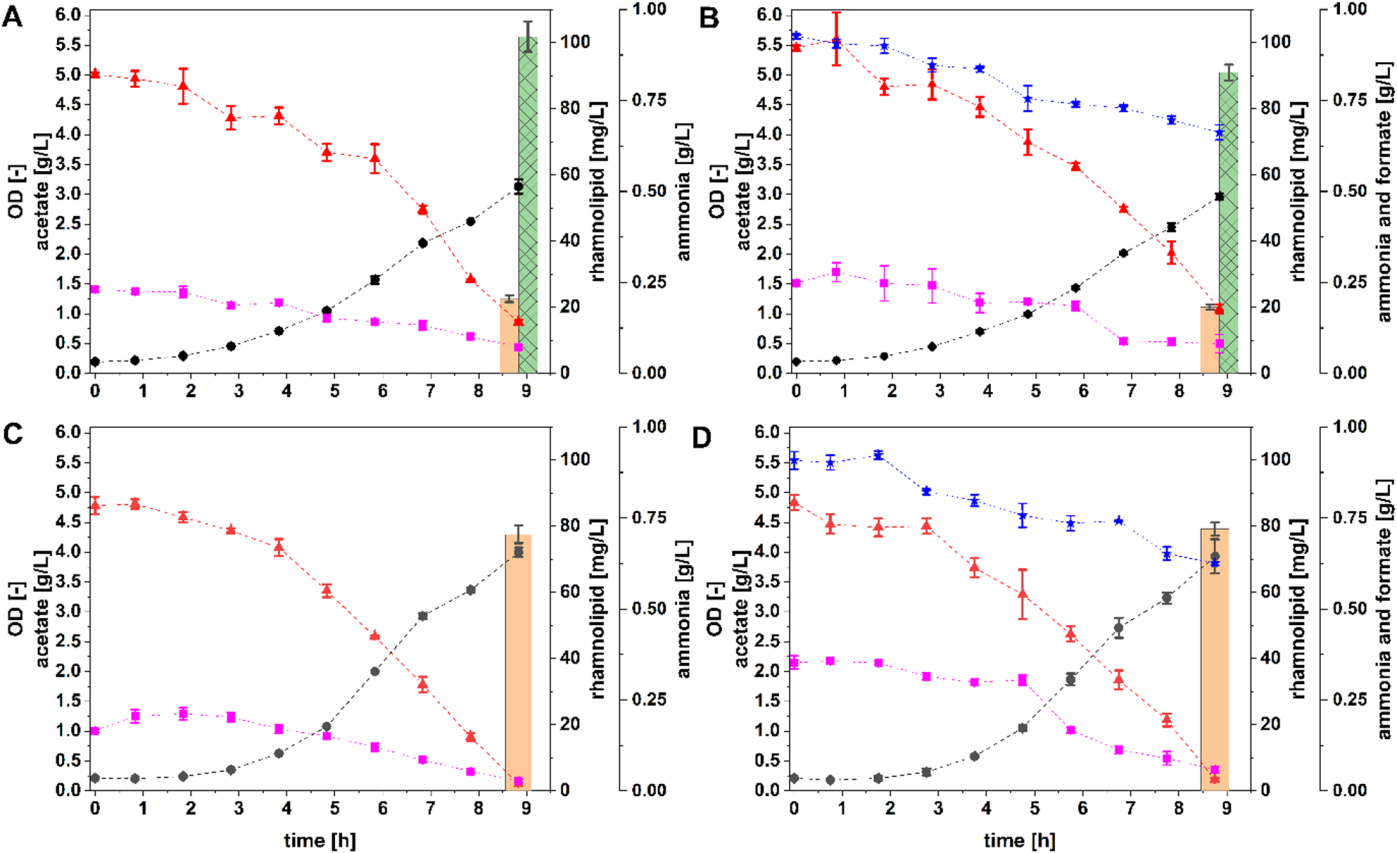
Co-feeding of rhamnolipid producers with acetate and formate. Acetate in red triangles, formate in blue asterisks, OD in black dots, ammonia in purple squares. Mono-rhamnolipid as brown bar, Di-rhamnolipid as green hatched bars. **A**, **B**: cultivation diagrams of *P. putida* JAG1; **C**, **D**: cultivation diagrams of *P. putida* JAG2

Taking the proteome analysis as imprint of bacterium’s metabolism, the results suggest less metabolic flux via pathways competing for rhamnolipid production when acetate is used as carbon source. For example, pathways of the Entner-Doudoroff are downregulated, which might prevent the generation, export and therefore, loss of carbon via 2-ketogluconate. Also, PhaG, the initial enzyme of polyhydroxyalkanoate (PHA) synthesis, was downregulated (log2(fc)=-1.55). The production of the polymer PHA is known to negatively impact RL synthesis, and is therefore knocked-out in several producer strains of RL and hydroxyalkanoyloxy alkanoic acids [40, 41]. Nevertheless, according to the calculated estimates, the yield of rhamnolipid on acetate appeared to be slightly lower than that observed with glucose. (Table 1). The reason for this presumably lies in the lower amount of NADPH that can be obtained from acetate. Rhamnolipid synthesis is strongly dependent on anabolic reactions that need NADPH, so for example the last reaction of the rhamnose pathway catalyzed by RmlD, or reactions of the *de-novo* fatty acid synthesis catalyzed by FabG and FabV. While NADPH regeneration happens in high rates in glucose metabolism [42], it relies on gluconeogenesis as well as activity of transhydrogenases when acetate is the carbon source. The latter can be assumed from increased abundance of the transhydrogenases SthA (log2(fc) = 1.75) and PntAB (log2(fc) = 0.47) (Fig. 1). An excess availability of NADH, combined with limited transhydrogenase-mediated NADPH regeneration under acetate-consuming conditions, may partly explain why formate supplementation did not measurably affect the performance of the rhamnolipid-producing strains (Fig. 3; Table 3). Overexpression of transhydrogenase genes could therefore be a strategy to increase the pool of NADPH and increase dependent anabolic reactions, as has been shown before for *Escherichia coli* [43]. Another reason for the lower yields obtained with acetate compared to glucose may be a reduced carbon flux, potentially limited by acetate uptake or intracellular conversion. Increased uptake rates of carbon can improve the productivity of the microbes [44]. In *P. putida* KT2440, overexpression of acetyl-CoA synthetase significantly increased the cell dry weight concentration, indicating that acetate activation may represent a limiting step for biomass formation on acetate [27, 45]. In the present proteome analysis, the enzyme ScpC showed the highest increase in abundance during growth on acetate. Overexpression of the *scpC*-gene or acetate transporters (such as *actP-I*) might therefore be rational strategies to enhance acetate assimilation [5]. In addition, the turnover rate of the TCA cycle could be increased by overexpression of citrate synthase gene *gltA* to rapidly convert acetyl-CoA [46]. Another approach to increase strain performance on acetate could be directed evolution, for example to increase resistance to higher extracellular acetate concentrations or to improve growth rates, as previously demonstrated [47].

## Conclusions

The utilization of acetate as a more sustainable alternative carbon source to glucose for microbial synthesis of value-added products will become increasingly important in the future. To date, the effect that acetate has on the bacterial physiology and metabolism, and comparative data to established carbon sources such as glucose, are scarce. The highly valuable biosurfactants rhamnolipids are, due to their versatile applications, of high interest for industrial application, and much effort has been made to optimize their production in safe heterologous microbial cell factories such as *Pseudomonas putida* KT2440. However, most research to date has concentrated on the production of mono-rhamnolipids and the utilization of glucose as a carbon source for this purpose. This study provides insights into the production of different rhamnolipid congeners and the impact of the carbon source on the product composition, focusing on acetate as future oriented sustainable alternative to glucose. A novel approach for acetate production with negative carbon footprint is the electrolysis of CO_2_ and water. In a model electrolysis medium derived from literature, all contained carbon components were consumed simultaneously and converted to biomass and rhamnolipid. This offers new opportunities for future studies focusing on the use of electrolytically produced acetate and similar small carbon compounds for biotechnological processes. In addition, the results of this study will hopefully encourage researchers to place greater emphasis on the production of the di-rhamnolipid congener. This is because, at least in the case of acetate as the carbon source, the conversion of carbon atoms into this congener appears to be more efficient compared to that of mono-rhamnolipids. Furthermore, comparative proteome analysis gives insights into the adjustments of *P. putida* KT2440 to cope with the different carbon sources and may evoke novel impulses for understanding and engineering the metabolism of the bacterium.

## Supplementary Information

Below is the link to the electronic supplementary material.

Additional file 1.

Additional file 2.

Additional file 3.

Additional file 4.

Additional file 5.

## Data Availability

Availability of data and materials: The mass spectrometry proteomics data will be deposited to the ProteomeXchange Consortium via the PRIDE partner repository with the dataset identifier PXD074559.
