# Supplementary material for: Acetate as alternative carbon source for production of mono- and di-rhamnolipids in Pseudomonas putida KT2440
Source: Microb Cell Fact. 2026 Jul 2;25:151. doi: 10.1186/s12934-026-03050-6 (PMC13330470; doi:10.1186/s12934-026-03050-6)
Supplement: Supplementary file 2 — Additional file 2. [file 12934_2026_3050_MOESM2_ESM.pdf]

Acetate as alternative carbon source for production of mono- and di-rhamnolipids in Pseudonomas putida KT2440

Authors: Jakob Grether, Sarah Leibinger, Christina Ramke, Philipp Hubel, Lisa Weber, Jens Pfannstiel, Elvio Henrique Benatto Perino, Rudolf Hausmann

Additional file 1

Full sequence of pCRA32 (*Δupp* counterselection plasmid for generation of *Pseudomonas putida* RAC10)

ctcgcgcgtttcggtgatgacggtgaaaacctctgacacatgcagctcccggagacggtcacagcttgtctgtaagcggatgccgggagcagaca
agcccgtcagggcgcgtcagcgggtgttggcgggtgtcggggctggcttaactatgcggcatcagagcagattgtactgagagtgcaccatatAGg
gtcaggcgtccttttgcttggtgccgaagatcttgtcaccggcatcacccaggcctggcacgatgtagccgtgctcgttcaggcgctggtcgatcgag
gcggtgtagatcttcacgtccgggtgggctttttccaccacctcgatgccttctggtgcggcgaccagcaccatggcgcgaatctctttgcagccgg
cctttttcagcaggtcgatggtggcaaccatcgagccgccggtggccagcatcgggtcgatgatcagggccaggcgctggttgatgtccggcgcga
gcttttccagataggtgtgggcttcgagggtttcttcgtttcgggcaacgccgacggcgctgaccttggcccccgggatcaggctgagcacgccgtc
gagcatgccgatgccggcgcgcaggatcggtactacggtgatcttcttgccggcgatttttttcaaccgagaccttgccacaccagccgtcgatctcg
taggtttcgaggggcaggtcctgggtggcttcatacgtcaggagcgcgccgacttcctgggcgagttcgcgaaaattcttggtgctgatatcggcacg
gcgcatcaggccaagcttgtggcggatcagcggatggcggatctcacgagtgggcataggggagggctccgaaaggcgggcaaaaaaaccgcg
ctagattaatctattcagcctgtgctgtcgtctggtcattctggacgttagtccataaatgcttgatctgtgacgagcggatgcgtacctttgcccgctttt
ccaaaatgctagccggctacgtatcgataagcttcacgctgccgcaagcactcagggcgcaagggctgctaaaggaagcggaacacgtagaaa
gccagtccgcagaaacggtgctgaccccggatgaatgtcagctactgggctatctggacaagggaaaacgcaagcgcaaagagaaagcaggta
gcttgcagtgggcttacatggcgatagctagactgggcggttttatggacagcaagcgaaccggaattgccagctggggcgccctctggtaaggttg
ggaagccctgcaaagtaaactggatggctttcttgccgccaaggatctgatggcgcaggggatcaagatctgatcaagagacaggatgaggatcgt
ttcgcatgattgaacaagatggattgcacgcaggttctccggccgcttgggtggagaggctattcggctatgactgggcacaacagacaatcggctg
ctctgatgccgccgtgttccggctgtcagcgcaggggcgcccggttctttttgtcaagaccgacctgtccggtgccctgaatgaactgcaggacgag
gcagcgcggctatcgtggctggccacgacgggcgttccttgcgcagctgtgctcgacgttgtcactgaagcgggaagggactggctgctattgggcg
aagtgccggggcaggatctcctgtcatctcaccttgctcctgccgagaaagtatccatcatggctgatgcaatgcggcggctgcatacgcttgatcc
ggctacctgcccattcgaccaccaagcgaaacatcgcatcgagcgagcacgtactcggatggaagccggtcttgtcgatcaggatgatctggacg
aagagcatcaggggctcgcgccagccgaactgttcgccaggctcaaggcgcgcatgcccgacggcgaggatctcgtcgtgacccatggcgatgc
ctgcttgccgaatatcatggtggaaaatggccgcttttctggattcatcgactgtggccggctgggtgtggcggaccgctatcaggacatagcgttggc
tacccgtgatattgctgaagagcttggcggcgaatgggctgaccgcttcctcgtgctttacggtatcgccgctcccgattcgcagcgcatcgccttct
atcgccttcttgacgagttcttctgagcgggactctggggttcgaaatgaccgaccaagcgacgcccaacctgccatcacgagatttcgattccac
cgccgccttctatgaaaggttgggcttcggaatcgttttccgggacgccggctggatgatcctccagcgcggggatctcatgctggagttcttcgccc
accccgggctcgatcccctcgcgagttggttcagctgctgcctgaggctggacgacctcgcggagttctaccggcagtgcaaatccgtcggcatcc
aggaaaccagcagcggctatccgcgcatccatgcccccgaactgcaggagtggggaggcacgatggccgctttggtcccgagaaaagccgctg
gccctatggggtgagcggctttttttgtttggctttgcCcgtgggcaactggaccatcacccctggcgtgcgcttcgagcacatcaataccgactggcg
cgaccgcccggtgctggacgccaacaaccgcccggtagccgagaagaagcgcagcatcaccagcaacgagcctttgccggcactgagcgtga
tgtaccacgtttccgatgcctggaaggtattcgccaactacgagacgtcgttcggtagcctgcagtacttccagctgggccagggcggttctggcaa
cagtacggccGatggcctggagccggaaaaagccaagacctacgaaatcggtacccgttatgacaatggcggctttgccggtgagctgacggcg
ttctacatcgacttcgatgatgagctgcagtacatcagcaacgacgtgggctggaccaacctcggtgcgaccaagcaccagggtatcgaggcttc
ggtgcgttatgacctggccgggctggacccgcgcctggacgggctgtcggtgagcggtggctacacctatacccgcgcgacctatgaaggggatat
tccagggttcaagggccgagacctgccgttctattcacgccaggtggccactgccggggtgcgttacgcagttaaccgctggGcctggaacctgg
acgcctatgcccaatcgaagcagcgtgcgccgggaaccgggatcaatgccgatggcagtttcaatggcgactacatcaccgagccgagtgctga
cgggcagtatggcgatattccaggctacgtgacctggcatgcccgtggcggttatgagttcgggccggacatgtccaacctgaagctggcggcggg
ggtgaagaacctgttcgacaagcagtacttcacccgctccagtgacaacaatgccggcctttacgtgggggagccgcggaccttctacgtgcagg
ccagtgttgggttttgatccgagactgtcggggccgctttgcggcccttcgctcgctcttCtcgagAAAAAaccggtTGTGCCGTAACTGC
AAGATCATCCGtcgcgaaggcgtcgtacgagtgatctgcagcgcggaaccgcgtcacaaacagcgccaaggctgagtgtgatctgcgcttca
aacccagcagctagtgtgctgctgggttgattattcgtttctacagcgatattatctcgcgccctatttcttggCTTCCGGGGCGTAGGTAGCc
TTAAGATTTTTGGGAGGTGTGAAATGCGGCGCGAAAGTCTGTTGGTATCGGTTTGCAAGGGCCTGCGGGTACATG

TCGAGCGCGTTGGGCAGGATCCCGGGCGCAGCACGGTGATGCTGGTCAACGGCGCGATGGCGACCACCGC
CTCGTTCGCCCGGACCTGCAAGTGCCTGGCCGAACATTTCAACGTGGTGCTGTTCGACCTGCCCTTCGCCGG
GCAGTCGCGTCAGCACAACCCGCAGCGCGGGTTGATCACCAAGGACGACGAGGTGGAAATCCTCCTGGCGC
TGATCGAGCGCTTCGAGGTCAATCACCTGGTCTCCGCGTCCTGGGGCGGTATCTCCACGCTGCTGGCGCTGTC
GCGCAATCCGCGCGGCATCCGCAGCTCGGTGGTGATGGCATTCGCCCCTGGACTGAACCAGGCGATGCTCG
ACTACGTCGGGCGGGCGCAGGCGCTGATCGAGCTGGACGACAAGTCGGCGATCGGCCATCTGCTCAACGAG
ACCGTCGGCAAATACCTGCCGCAGCGCCTGAAAGCCAGCAACCATCAGCACATGGCTTCGCTGGCCACCGG
CGAATACGAGCAGGCGCGCTTTCACATCGACCAGGTGCTGGCGCTCAACGATCGGGGCTACTTGGCTTGCCT
GGAGCGGATCCAGAGCCACGTGCATTTCATCAACGGCAGCTGGGACGAATACACCACCGCCGAGGACGCCC
GCCAGTTCCGCGACTACCTGCCGCACTGCAGTTTCTCGCGGGTGGAGGGCACCGGGCATTTCCTCGACCTGG
AGTCCAAGCTGGCAGCGGTACGCGTGCACCGCGCCCTGCTCGAGCACCTGCTGAAGCAACCGGAGCCGCA
GCGGGCGGAACGCGCGGCGGGATTCCACGAGATGGCCATCGGCTACGCCTGAACCCTTGACCTGCGAAGA
CCCGGCCTGGCCGGGCTTTGCGGTTGCATAACGCACGGAGTAGCCCCATGCACGCCATCCTCATCGCCATC
GGCTCGGCCGGCGACGTATTTCCCTTCATCGGCCTGGCCCGGACCCTGAAACTGCGCGGGCACCGCGTGAG
CCTCTGCACCATCCCGGTGTTTCGCGACGCGGTGGAGCAGCACGGCATCGCGTTCGTCCCGCTGAGCGACG
AACTGACCTACCGCCGGACCATGGGCGATCCGCGCCTGTGGGACCCCAAGACGTCCTTCGGCGTGCTCTGG
CAAGCCATCGCCGGGATGATCGAGCCGGTCTACGAGTACGTCTCGGCGCAGCGCCATGACGACATCGTGGTG
GTCGGCTCGCTATGGGCGCTGGGCGCACGCATCGCTCACGAGAAGTACGGGATTCCCTACCTGTCCGCGCAG
GTCTCGCCATCGACCCTGTTGTCGGCGCACCTGCCGCCGGTACACCCCAAGTTCAACGTGCCCGAGCAGATG
CCGCTGGCGATGCGCAAGCTGCTCTGGCGCTGCATCGAGCGCTTCAAGCTGGATCGCACCTGCGCGCCGGA
GATCAACGCGGTGCGCCGCAAGGTCGGCCTGGAAACGCCGGTGAAGCGCATCTTCACCCAATGGATGCATTC
GCCGCAGGGCGTGGTCTGCCTGTTCCCGGCCTGGTTCGCGCCGCCCCAGCAGGATTGGCCGCAACCCCTG
CACATGACCGGCTTCCCGCTGTTCGACGGCAGTATCCCGGGGACCCCGCTCGACGACGAACTGCAACGCTTT
CTCGATCAGGGCAGCCGGCCGCTGGTGTTCACCCAGGGCTCGACCGAACACCTGCAGGGCGACTTCTACGC
CATGGCCCTGCGCGCGCTGGAACGCCTCGGCGCGCGTGGGATCTTCCTCACCGGCGCCGGCCAGGAACC
GCTGCGCGGCTTGCCGAACCACGTGCTGCAGCGCGCCTACGCGCCACTGGGAGCCTTGCTGCCATCGTGC
GCCGGGCTGGTCCATCCGGGCGGTATCGGCGCCATGAGCCTAGCCTTGGCGGCGGGGGTGCCGCAGGTGC
TGCTGCCCTGTGCCCACGACCAGTTCGACAATGCCGAACGGCTGGTCCGGCTCGGCTGCGGGATGCGCCTG
GGCGTGCCGTTGCGCGAGCAGGAGTTGCGCGGGGCGCTGTGGCGCTTGCTCGAGGACCCGGCCATGGCGG
CGGCCTGTCGGCGTTTCATGGAATTGTCACAACCGCACAGTATCGCTTGCGGTAAAGCGGCCCAGGTGGTCGA
ACGTTGTCATAGGGAGGGGGATGCTCGATGGCTGAAGGCTGCGTCCTGAGTTTAAACttacccgcgaaggggcgcaaa
gcgcccccggcgattacgaaaccaccgcctcggccggcgaaacgatactgctcttcgcccccccgcgatctccccgagctcaggtacgcggcaa
tcgactcctgcgtcacctcgcccaggaacacctgctcggcatccaacactggcGaccacgcgcggttgaactcgtacatgcgcgacaacagga
tgcgcaaatgctcatcgtgcgacgcggtggcattgaacggccgcaggaaatcgccacacgtcccttgctggcggtgcatgtcgcgccggcgcaca
tagcccagcgccttgttctgcgcatcggtcaccaccacgtagcggcggtcgtgctcatcgagcagctccagcgcgtcgcccaccggtgtttccggg
ctcaccgacggcgcgttgtccgcCgcatcctcggcacgcaccagcaacaggcgtttgagggtgctgtcctggcccacaaagttgctgacgaaat
catccgccgggtgcgccaacagggtatccgggtgatccagctgcaacagcttgccggcacggaaaatggcaatcttgtcgcccagtttgatcgctt
cgtcgatgtcgtggctgaccatgattaccgtcttgttcagcgcccgctgcatttcgaagaactcgttctggatcatctcgcggttgatcgggtcgaccg
cgccgaacggctcgtccatcaacagcacgggtgcatctgctgccagggcgcgaatcacgccgatacgctgttgctggccgccagacagctcgcg
tgggtagcgttgcaggtactgcttgggttcgagcttgatcatgtgcatcagctcgcgagcccgctcgtggcatttctgtttgtcccagccaagcaggcg
cgggaccacggtaatgttctcctcgatggtcatgttggggaacagaccgatctgctgaatcacatagccgatgtgccgTTAgacctgcaggcatgc
aagcttggcgtaatcatggtcatagctgtttcctgtgtgaaattgttatccgctcacaattccacacaacatacgagccggaagcataaagtgtaaag
cctggggtgcctaatgagtgagctaactcacattaattgcgttgcgctcactgcccgctttccagtcgggaaacctgtcgtgccagctgcattaatga
atcggccaacgcgcggggagaggcggtttgcgtattgggcgctcttccgcttcctcgctcactgactcgctgcgctcggtcgttcggctgcggcgag
cggtatcagctcactcaaaggcggtaatacggttatccacagaatcaggggataacgcaggaaagaacatgtgagcaaaaggccagcaaaagg
ccaggaaccgtaaaaaggccgcgttgctggcgtttttccataggctccgcccccctgacgagcatcacaaaaatcgacgctcaagtcagaggtg
gcgaaacccgacaggactataaagataccaggcgtttccccctggaagctccctcgtgcgctctcctgttccgaccctgccgcttaccggatacc
tgtccgcctttctcccttcgggaagcgtggcgctttctcatagctcacgctgtaggtatctcagttcggtgtaggtcgttcgctccaagctgggctgtgtg
cacgaaccccccgttcagcccgaccgctgcgccttatccggtaactatcgtcttgagtccaacccggtaagacacgacttatcgccactggcag
cagccactggtaacaggattagcagagcgaggtatgtaggcggtgctacagagttcttgaagtggtggcctaactacggctacactagaaggacag

tatttggtatctgcgctctgctgaagccagttaccttcggaaaaagagttggtagctcttgatccggcaaacaaaccaccgctggtagcggtggtttttttgtttgcaagcagcagattacgcgcagaaaaaaaggatctcaagaagatcctttgatcttttctacggggtctgacgctcagtggaacgaaaactcacgttaagggattttggtcatgagattatcaaaaaggatcttcacctagatccttttaaattaaaaatgaagttttaaatcaatctaaagtatatatgagtaaacttggtctgacagttaccaatgcttaatcagtgaggcacctatctcagcgatctgtctatttcgttcatccatagttgcctgactccccgtcgtgtagataactacgatacgggagggcttaccatctggccccagtgctgcaatgataccgcgagacccacgctcaccggctccagatttatcagcaataaaccagccagccggaagggccgagcgcagaagtggtcctgcaactttatccgcctccatccagtctattaattgttgccgggaagctagagtaagtagttcgccagttaatagtttgcgcaacgttgttgccattgctacaggcatcgtggtgtcacgctcgtcgtttggtatggcttcattcagctccggttcccaacgatcaaggcgagttacatgatcccccatgttgtgcaaaaaagcggttagctccttcggtcctccgatcgttgtcagaagtaagttggccgcagtgttatcactcatggttatggcagcactgcataattctcttactgtcatgccatccgtaagatgcttttctgtgactggtgagtactcaaccaagtcattctgagaatagtgtatgcggcgaccgagttgctcttgcccggcgtcaatacgggataataccgcgccacatagcagaactttaaaagtgctcatcattggaaaacgttcttcggggcgaaaactctcaaggatcttaccgctgttgagatccagttcgatgtaacccactcgtgcacccaactgatcttcagcatcttttactttcaccagcgtttctgggtgagcaaaaacaggaaggcaaaatgccgcaaaaaagggaataagggcgacacggaaatgttgaatactcatactcttcctttttcaatattattgaagcatttatcagggttattgtctcatgagcggatacatatttgaatgtatttagaaaaataaacaaataggggttccgcgcacatttccccgaaaagtgccacctgacgtctagaagggacagtgaagaaggaacacccgctcgcgggtgggcctacttcacctatcctgccccggctgacgtctaagaaaccattattatcatgacattaacctataaaaataggcgtatcacgaggccctttcgt

Table S 1 List of primers for cloning of the plasmids pJG-rhlCAB, pTn7-rhlCAB and pTn7-rhlAB

Primer name	Sequence (5’-3’)	Purpose
**rhlC_fw**	AAACAGCTATCGCTGCCACGCTAGGCCTTGGCCTTGC	Cloning of pJG-rhlCAB, amplification of *rhlC*
**rhlC_rv**	AGTCATCGGCTACGCGTTACGGGAGAAGAACGATCATGG	
**pJG-rhlAB-backbone_fw**	AAGGCCAAGGCCTAGCGTGGCAGCGATAGCTGTTTGCC	Cloning of pJG-rhlCAB, plasmid backbone(BB) amplification
**pJG-rhlAB_backbone_rv**	TGATCGTTCTTCTCCCGTAACGCGTAGCCGATGACTGA	
**Tn7_insert_fw**	CCTGCAGGTCGACTCTAGAGGATCCAGCTCTTGACAAGGTCG	Amplification of SynPro8-rhlCAB or SynPro8_rhlAB
**Tn7_insert_rv**	CGCGAATTCGAGCTCGGTACGCTCACTCATTAGGCACC	
**pTn7-M_backbone_fw**	GTACCGAGCTCGAATTC	Cloning of pTn7-rhlCAB and pTn7-rhlAB, BB amplification
**pTn7-M_backbone_rv**	GATCCTCTAGAGTCGACC	
**attTn7_seq_fw**	AGTCAGAGTTACGGAATTGTAGG	Sequencing
**attTn7_seq_rv**	TTACGTGGCCGTGCTAAAGGG	Sequencing

Table S 2 List of plasmids and strains generated or used in the study

Strain/plasmid name	Genotype/plasmid description	Reference
P. putida* KT2440 *Δupp	*P. putida* KT2440 derivative carrying a deletion of the *upp* gene	Graf and Altenbuchner, 2011
***P. aeruginosa* PAO1 (genomic DNA)**	Wildtype, DSM 19880	Stover, Pham 2000
***P. putida* RAC10**	*P. putida* KT2440 Δ*upp*, P_{rpsM}::*rhlAB*	This study

P. putida **JAG1**	RAC10 $P_{SynPro8oT}$::*rhlCAB*	This study
P. putida **JAG2**	RAC10 $P_{SynPro8oT}$::*rhlAB*	This study
**pJOE6261.2**	Markerless chromosomal integration via the *upp* counterselection system	Graf and Altenbuchner, 2011
**pCRA32**	pJOE6261.2 derivative carrying P_{rpsM}::*rhlAB*	This study
**pJG-rhlCAB**	pJG-rhlAB derivative $P_{SynPro8oT}$::*rhlCAB*	This study
**pJG-rhlAB**	$P_{SynPro8oT}$::*rhlAB*	Grether et al., 2025
**pTn7-M**	Mini-Tn7 delivery vector for chromosomal integration at the attTn7 site	Zobel et al., 2015
**pTn7-rhlCAB**	pTn7-M derivative carrying $P_{SynPro8oT}$::*rhlCAB*	This study
**pTn7-rhlAB**	pTn7-M derivative carrying $P_{SynPro8oT}$::*rhlAB*	This study
