# Supplementary material for: Acetate as alternative carbon source for production of mono- and di-rhamnolipids in Pseudomonas putida KT2440
Source: Microb Cell Fact. 2026 Jul 2;25:151. doi: 10.1186/s12934-026-03050-6 (PMC13330470; doi:10.1186/s12934-026-03050-6)
Supplement: Supplementary file 3 — Additional file 3. [file 12934_2026_3050_MOESM3_ESM.pdf]

Acetate as alternative carbon source for production of mono- and di-rhamnolipids in Pseudonomas putida KT2440

Authors: Jakob Grether, Sarah Leibinger, Christina Ramke, Philipp Hubel, Lisa Weber, Jens Pfannstiel, Elvio Henrique Benatto Perino, Rudolf Hausmann

Additional file 4

Rhamnolipid standard calibration curves

For rhamnolipid calibration, different amounts of derivatized rhamnolipids are plated and developed on HPTLC F254 plates. Peak measurement is based on fluorescence extinction of the attached bromophenacyl group.

Table 1: Data of RL calibration for the rhamnolipid quantification using glucose or acetate, respectively, as carbon source for fermentation. Different amounts of rhamnolipid were plated and the peak area measured.

	Peak area [a.u.]	
Rhamnolipid plated [µg]	glucose as carbon source	acetate as carbon source
0.075	996.4	938.2
0.125	1509.9	1407.1
0.25	2465.7	2419.9
0.625	4895.6	4816.5
1	7005.7	6915.1
1.5	8698.6	8772.4
2	10464.2	10508.2
2.5	11433.4	11591.4

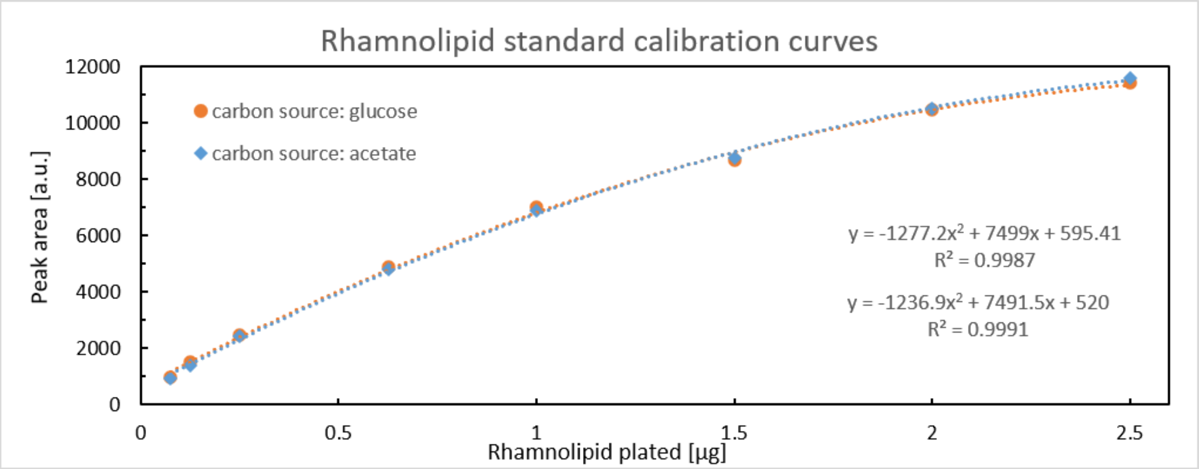

Figure 1: Plots of rhamnolipid calibration curves. Peak areas obtained in HPTLC measurement were plotted against the plated rhamnolipid quantity to obtain calibration curves with $R^2 > 0.995$. To demonstrate the reproducibility of the method, calibration curves applied for quantification of rhamnolipids produced during growth on glucose (orange circles) and acetate (blue diamonds) are presented.

Rhamnolipid congener separation via HPTLC

Due to their different hydrophobicity, mono-rhamnolipids and di-rhamnolipids can be separated into two clearly distinguishable peaks by HPTLC. This allows separate quantification of the congeners. The following figure represents the peak separation:

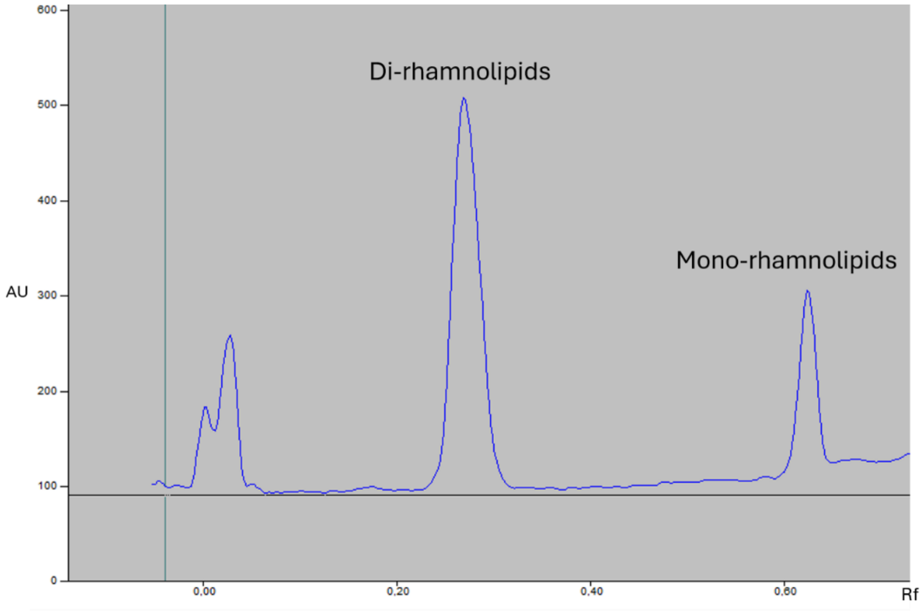

Figure 2: HPTLC Chromatogram of rhamnolipid congeners. The chromatogram shows the separation of rhamnolipid congeners obtained by HPTLC analysis. The main peaks were assigned to di-rhamnolipids and mono-rhamnolipids according to their retention behavior, with di-rhamnolipids being more hydrophilic than mono-rhamnolipids.
